# B Cell Modulation Strategies in Autoimmune Diseases: New Concepts

**DOI:** 10.3389/fimmu.2018.00622

**Published:** 2018-04-13

**Authors:** Philippe Musette, Jean David Bouaziz

**Affiliations:** ^1^Dermatology Department, INSERM U976, Rouen University Hospital, Rouen, France; ^2^Dermatology Department, INSERM U976, Saint Louis University Hospital, Paris, France

**Keywords:** autoimmunity, autoantibodies, B cell, B cell depletion, regulatory B cell

## Abstract

B cells are major effector cells in autoimmunity through antibody production, T cell help and pro-inflammatory cytokine production. Major advances have been made in human B cell biology knowledge using rituximab and type II new anti-CD20 antibodies, anti-CD19 antibodies, anti-CD22 antibodies, autoantigen specific B cell depleting therapy (chimeric antigen receptor T cells), and B cell receptor signaling inhibition (Bruton’s tyrosine kinase inhibitors). However, in certain circumstances B cell depleting therapy may lead to the worsening of the autoimmune disease which is in accordance with the existence of a regulatory B cell population. Current concepts and future directions for B cell modulating therapies in autoimmune diseases with a special focus on pemphigus are discussed.

## Introduction

B cells were primary identified for their key role as enhancers of the immune response in autoimmunity, because they give rise to autoantibody producing plasma cells and promote CD4^+^ T cell responses by antigen presentation. The B cells bearing these functions are usually considered as effector B cells. Recently published studies indicate that B cells can also act as negative sensors of the immune response in autoimmunity, these regulatory properties are mainly attributed to the recently identified interleukin 10 (IL-10) regulatory B cell compartment (Breg) (1–3). New therapies target these B cell populations with drugs directed against B cell surface markers (CD20, CD22), activating factors (BAFF, TACI), or cytokines (IL-6, TNFα, IFNα) (4, 5). The most focused strategy to target B cells in autoimmune diseases would be to specifically remove autoreactive effector B cells, and amplify autoantigen driven Bregs, while maintaining immune surveillance. Such a strategy is difficult to achieve especially because antigen specific targeting is challenging and, the relative contribution of B cells to the pathogenesis of autoimmune disease might differ considerably from one disease to another.

Autoreactive B cells give rise to autoreactive plasma cells whose pathogenicity might be direct through production of IgG^+^ autoantibodies that bind to specific target molecules (e.g., acetylcholine receptor on the motor end plate in myasthenia gravis; desmoglein 1 and 3 on keratinocyte in pemphigus) or through the formation of immune complexes in tissues that locally activate the complement cascade. B cells are also important effector cells in autoimmune diseases because they regulate lymphoid tissue structure, contribute to antigen presentation and costimulation (6), regulate dendritic cell function and pathways of T helper cell differentiation, and release inflammatory cytokines including IL-8, IL-6, LT-α, and TNF-α (7–10). Our review will focus on B cell therapies in various autoimmune disorders with a special focus on pemphigus (an autoimmune blistering skin disease). Table 1 summarizes recent studies in pemphigus that target B cells or their pathogenic antibodies.

**Table 1:** Current ongoing studies in pemphigus that target B cells or their pathogenic antibodies (excluding studies with rituximab).

A Long-Term Extension Study of Ofatumumab (**type I anti-CD20**) Injection for Subcutaneous Use in Subjects With Pemphigus Vulgaris *ClinicalTrials.gov identifier: NCT02613910*
Efficacy and Safety of Ofatumumab (**type I anti-CD20**) in Treatment of Pemphigus Vulgaris *ClinicalTrials.gov identifier: NCT01920477*
Study of Efficacy and Safety of VAY736 (**anti-BAFF-R**) in Patients With Pemphigus Vulgaris *ClinicalTrials.gov identifier: NCT01930175*
A Study to Evaluate the Safety, PD, PK and Efficacy of ARGX-113 (**human IgG1-derived Fc fragment that binds to FcRn**) in Patients With Pemphigus *ClinicalTrials.gov identifier: NCT03334058*
A Safety Study of SYNT001 (**A Humanized IgG4 Monoclonal Antibody That Disrupts the Interaction of FcRn and IgG**) in Subjects With Pemphigus (Vulgaris or Foliaceus) *ClinicalTrials.gov identifier: NCT03075904*
A Study of PRN1008 (**Bruton’s tyrosine kinase inhibitor**) in Adult Patients With Pemphigus Vulgaris *ClinicalTrials.gov identifier: NCT02704429*

*Bold represents mechanisms of action of the drug*.

*FcRn, neonatal Fc Receptors*.

*New therapies seek to disrupt the IgG–FcRn interaction to increase the clearance of pathogenic IgG antibodies from the body*.

## Anti-CD20 mAb

B cell depletion using type 1 anti-CD20 monoclonal antibodies (rituximab, ofatumumab, ocrelizumab) has shown varying degrees of efficacy in some human autoimmune diseases ranging from dramatic efficacy to sometimes worsening of symptoms (4, 11). Rituximab has been proved to be highly efficient in rheumatoid arthritis, pemphigus (12, 13), granulomatosis with polyangiitis, and microscopic polyangiitis (14). Ocrelizumab was recently proved to be efficient and FDA approved in relapsing multiple sclerosis (15, 16).

**Figure 1:**
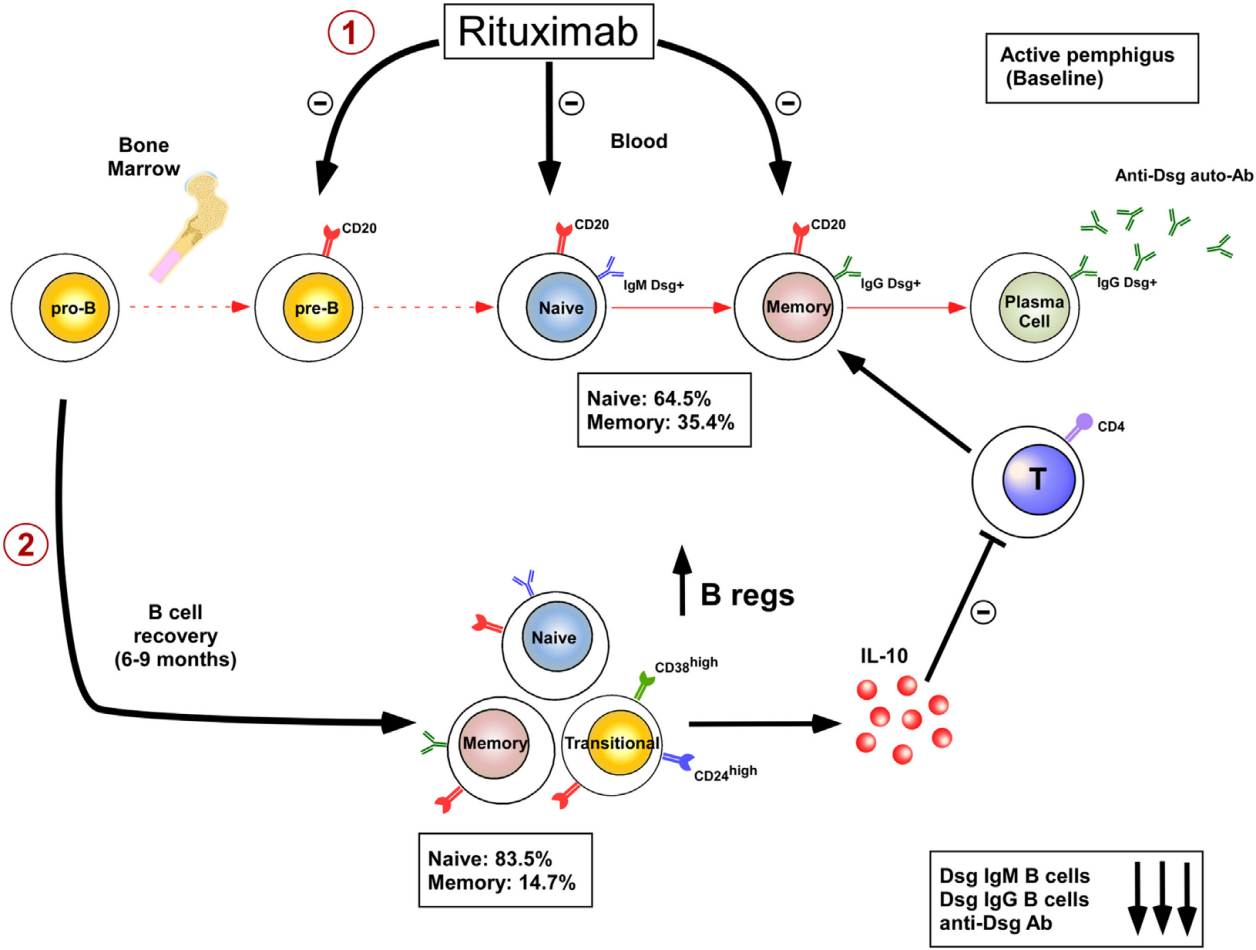
Dual mechanisms of B cell depletion: 1: elimination of autoreactive B cells; 2: induction of regulatory B cells.

The limited efficacy or adverse effects of B cell depleting therapies in some diseases may be partially explained by the fact that rituximab also depletes the Breg compartment. IL-10-producing Bregs were first identified in mice and shown to downregulate immune response and inflammation, making them probably instrumental for maintenance of self tolerance. Numerous recent studies have also characterized IL-10-producing Bregs in humans and have started to decipher their phenotype and mode of suppression. The cell surface phenotype of human Bregs is mainly composed of CD24^high^CD27^+^ B cell subpopulation (17, 18) and CD24^high^CD38^high^ transitional B cell subpopulation (19). Mechanisms of suppression include inhibition of CD4^+^ T proliferation, associated with induction and expansion of regulatory T cells, inhibition of Th1 differentiation, and also suppression of monocyte activation. The decreased frequency and/or decreased suppressive activity of Bregs have been recently shown in patients with lupus (19), immune thrombocytopenia (20), rheumatoid arthritis (21), ANCA-associated vasculitis (22), pemphigus (23), and systemic sclerosis (24). In addition an increase in Bregs is associated with better prognosis or complete remission in certain autoimmune diseases (25, 26). B cell depletion therapy using rituximab in systemic lupus erythematosus (SLE) (26, 27) and pemphigus (25) patients may also promote the emergence of a functional transitional Breg pool upon B cell reconstitution which may contribute to the long-term efficacy of rituximab in these diseases. Indeed rituximab induces a prolonged and continuous repopulation with naive B cells with a new repertoire (25, 28) after the initial B-cell depletion, whereas the reappearance of memory B cells is markedly delayed (25, 26). This blockage of B cell maturation is associated with a blockage of the auto-reactive IgM to IgG class switching process (25). Finally, B cell depletion induces a two step mechanism of immunosupression by eliminating the autoreactive B cells involved in the production of pathogenic IgG^+^ autoantibodies (28, 29) and by promoting the appearance of Bregs (25) (Figure 1). Rituximab therapy may, however, decrease the humoral immune response to recall antigens (30) which may increase the risk of patient infection including hepatitis B reactivation and progressive multifocal leukoencephalopathy (polyomavirus JC), whereas long-term anti tetanus and anti-pneumococcal antibody response is maintained (25). It also seems conceivable that, given the role of B cells in generating antitumor responses (31), B cell depletion using rituximab may contribute to long-term expansion of uncontrolled tumor cells (32). B cell depletion therapy using rituximab may be particularly efficient in pemphigus because it depletes memory B cells that give rise to short-lived plasma cells that produce pathogenic autoantibodies. Rituximab preserves long-lived plasma cells that may be more important for “natural” protection against infection. Pathogenic autoantibodies may be the result of a temporary breakdown of immune tolerance giving rise to pathogenic B cells clones that are abrogated using rituximab (33). B cell-activating factor (BAFF) and a proliferation-inducing ligand are important B cell differentiation and survival factors. Their targeting could also be interesting for pemphigus treatment.

A new class of “type II” anti-CD20 antibodies (obinutuzumab) that induce little complement-dependent cytotoxicity but induce signaling-dependent B cell death is under development (34). This type II class is particularly interesting because, contrary to rituximab, it may be more efficient at depleting organ resident B cells.

New strategies will require specific targeting of the pathogenic effector functions of B cells and if possible the promotion of their regulatory function without modifying B cell dependent immune surveillance.

## Anti-CD19 and Anti-CD22 mAbs

Anti-CD20 mAbs efficiently deplete mature naive and memory B cells, inhibit the development of short-lived plasma cells, but spare long-lived plasma cells and previously produced protective antibodies acquired during infection or vaccination. Contrary to CD20, CD19 is expressed from early B cell development (pro-B stage) to the last differentiation stage including plasma cells. These features make CD19 an attractive target for B cell depletion. MEDI-551 (inebilizumab) is a human IgG1 anti-CD19 mAb that was developed for the treatment of multiple sclerosis (35). Interestingly, bispecific antibodies (blinatumomab) that redirect T cells to CD19 have been developed and have shown efficacy for the treatment of acute lymphoblastic leukemia (36). Such bispecific antibodies that target leukemic and normal B cells could be an interesting approach for the treatment of autoimmune diseases in the future.

Epratuzumab is a humanized monoclonal antibody that binds to the glycoprotein CD22 (an inhibitory C-type lectin) expressed on mature and malignant B cells. Epratuzumab has completed phase III trials for the treatment of SLE (37). Although endpoints were not reached in this study, the drug was well tolerated. Epratuzumab diminishes B-cell receptor activation (38) and induces only partial B-cell depletion (39).

## Antigen Specific B Cell Depletion Using Chimeric Autoantibody Receptor (CAAR) T Cells

**Figure 2:**
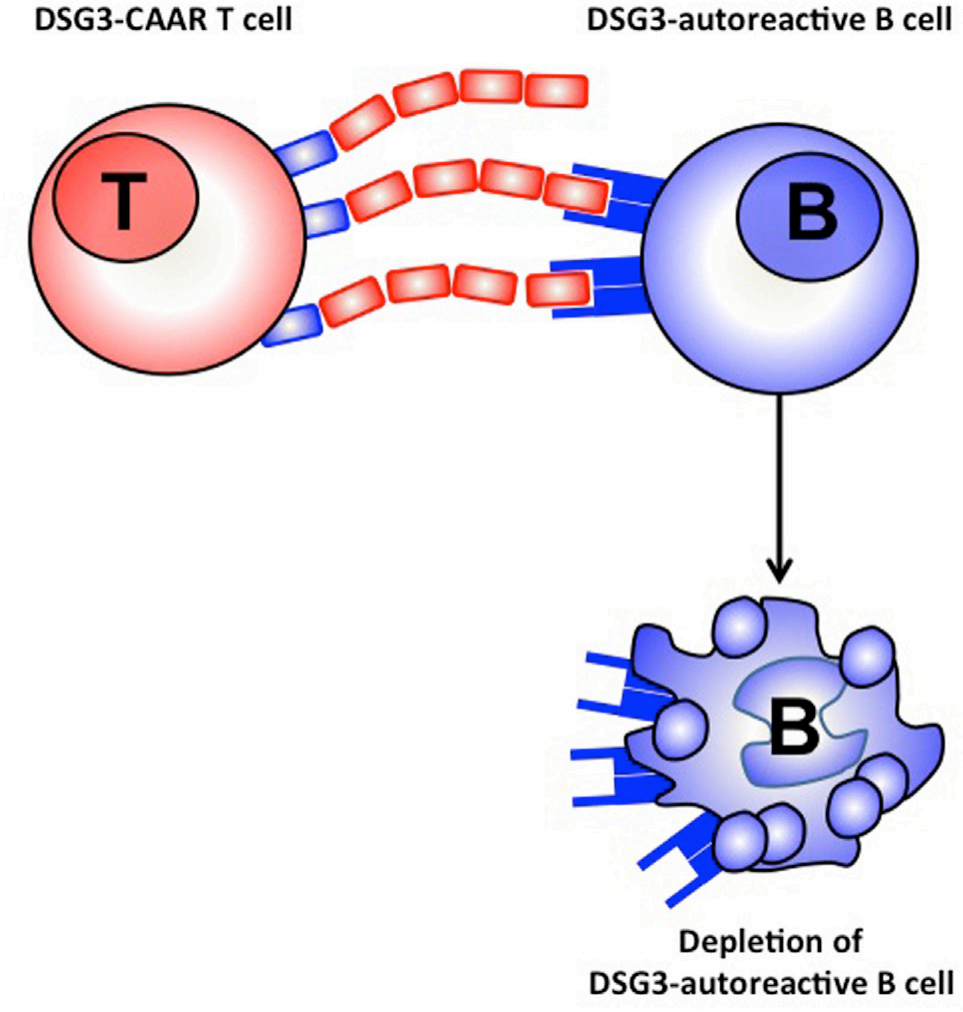
Chimeric auto antibody receptor (CAAR) T cell.

Targeting antigen specific immune response represents a very interesting strategy. In this respect a promising strategy has been developed for the treatment of pemphigus vulgaris, a blistering autoimmune skin disease, in which autoimmune B cells produce autoantibodies against the desmoglein 1 and 3 antigens present in the skin. Until recently, different approaches including immunoabsorption of Dsg autoantibodies *ex vivo* (40), or anti idiotype antibodies directed against Dsg3 specific autoantibodies (41) were developed without clinical impact. Recently, alive T cells that express a chimeric antigen receptor (CAR) composed of an antibody structure fused to the CD3 signaling domain engineered to recognize tumor-associated antigens have shown remarkable efficacy in B cell leukemia (42). Moreover CAR T cells are able to proliferate and expand *in vivo*. A new approach is now being developed with a CAAR including truncated fragments of the Dsg3 extracellular domain fused to CD137/CD3 signaling domains expressed on T cells. This CAAR is able to recognize autoantibodies against Dsg3 fixed on the B cell membrane (Figure 2). CAAR T cells have shown efficacy in eliminating anti-Dsg3 specific B cells *in vitro* and in a pemphigus mice model (43).

## Inhibition of B Cell Receptor Signaling

Activation of the B cell receptor induces downstream signaling of multiple kinases crucial for B cell activation. Among them, Lyn, Syk, PI3K, and Bruton’ tyrosine kinase (BTK) are potential therapeutic targets for autoreactive B cell silencing and depletion. Among the potential drugs, ibrutinib, a pharmacological BTK inhibitor has been developed and licensed for the treatment of chronic lymphocytic leukemia. Targeting BTK using ibrutinib may be an interesting approach to treat autoimmune diseases. Indeed, transgenic mice overexpressing BTK in B cells manifested SLE-like autoimmune disease involving kidneys, lungs, and salivary glands (44). Recently, ibrutinib has shown efficacy in chronic graft versus host disease the physiopathology of which involves allo-reactive B and T cells. Based on these results, ibrutinib was approved in the US for treatment of adult patients with GVHD after failure of one or more lines of systemic therapy (45). A new BTK inhibitor known as PRN1008 is under evaluation for the treatment of pemphigus in humans (ClinicalTrials.gov Identifier: NCT02704429).

## Conclusion

Much progress has been made in depleting circulating and resident B cells present in inflamed tissue and secondary lymphoid organs. An ideal strategy in the future will require specific targeting of the pathogenic effector functions of B cells and if possible the promotion of their regulatory function without modifying B cell-dependent immune surveillance. More targeted therapies on specific B cell populations and functions will allow better patient management.

## Author Contributions

PM and JDB contributed equally to the manuscript.

## Conflict of Interest Statement

The authors declare that the research was conducted in the absence of any commercial or financial relationships that could be construed as a potential conflict of interest.

## Abbreviations

CAR, chimeric antigen receptor; CAAR, chimeric autoantibody receptor; BTK, Bruton’s tyrosine kinase.
